# Bariatric surgery-induced weight loss and associated genome-wide DNA-methylation alterations in obese individuals

**DOI:** 10.1186/s13148-022-01401-9

**Published:** 2022-12-18

**Authors:** Fazlur Rahman Talukdar, David Israel Escobar Marcillo, Ruhina Shirin Laskar, Alexei Novoloaca, Cyrille Cuenin, Paolo Sbraccia, Lorenza Nisticò, Valeria Guglielmi, Tarik Gheit, Massimo Tommasino, Eugenia Dogliotti, Paola Fortini, Zdenko Herceg

**Affiliations:** 1 Epigenomics and Mechanisms Branch, International Agency for Research On Cancer (IARC), 150 Cours Albert Thomas, Lyon, France; 2 Early Detection, Prevention, and Infections Branch, International Agency for Research On Cancer (IARC), 150 Cours Albert Thomas, Lyon, France; 3 Section of Mechanisms, Biomarkers and Models, Dept Environment and Health, Istituto Superiore Di Sanità, Viale Regina Elena, No. 299, 00161 Rome, Italy; 4 Nutrition and Metabolism Branch, International Agency for Research On Cancer (IARC), 150 Cours Albert Thomas, Lyon, France; 5 Obesity Center-Internal Medicine Unit, Department of Systems Medicine, University of Rome Tor Vergata, 00133 Rome, Italy; 6 Centre for Behavioral Sciences and Mental Health, Istituto Superiore Di Sanità, Viale Regina Elena, No. 299, 00161 Rome, Italy; 7 IRCCS, Istituto Tumori Giovanni Paolo ll, Bari, Italy

**Keywords:** Obesity, Bariatric surgery, Epigenetics, DNA methylation, Weight loss, Epigenetic clock

## Abstract

**Background:**

Obesity is a multifactorial and chronic condition of growing universal concern. It has recently been reported that bariatric surgery is a more successful treatment for severe obesity than other noninvasive interventions, resulting in rapid significant weight loss and associated chronic disease remission. The identification of distinct epigenetic patterns in patients who are obese or have metabolic imbalances has suggested a potential role for epigenetic alterations in causal or mediating pathways in the development of obesity-related pathologies. Specific changes in the epigenome (DNA methylome), associated with metabolic disorders, can be detected in the blood. We investigated whether such epigenetic changes are reversible after weight loss using genome-wide DNA methylome analysis of blood samples from individuals with severe obesity (mean BMI ~ 45) undergoing bariatric surgery.

**Results:**

Our analysis revealed 41 significant (Bonferroni *p* < 0.05) and 1169 (false discovery rate *p* < 0.05) suggestive differentially methylated positions (DMPs) associated with weight loss due to bariatric surgery. Among the 41 significant DMPs, 5 CpGs were replicated in an independent cohort of BMI-discordant monozygotic twins (the heavier twin underwent diet-induced weight loss). The effect sizes of these 5 CpGs were consistent across discovery and replication sets (*p* < 0.05). We also identified 192 differentially methylated regions (DMRs) among which *SMAD6* and *PFKFB3* genes were the top hypermethylated and hypomethylated regions, respectively. Pathway enrichment analysis of the DMR-associated genes showed that functional pathways related to immune function and type 1 diabetes were significant. Weight loss due to bariatric surgery also significantly decelerated epigenetic age 12 months after the intervention (mean =  − 4.29; *p* = 0.02).

**Conclusions:**

We identified weight loss-associated DNA-methylation alterations targeting immune and inflammatory gene pathways in blood samples from bariatric-surgery patients. The top hits were replicated in samples from an independent cohort of BMI-discordant monozygotic twins following a hypocaloric diet. Energy restriction and bariatric surgery thus share CpGs that may represent early indicators of response to the metabolic effects of weight loss. The analysis of bariatric surgery-associated DMRs suggests that epigenetic regulation of genes involved in endothelial and adipose tissue function is key in the pathophysiology of obesity.

**Supplementary Information:**

The online version contains supplementary material available at 10.1186/s13148-022-01401-9.

## Introduction

Obesity is a multifactorial and chronic disease that adversely affects human health worldwide. Generally, people having a body mass index (BMI) of 25–30 kg/m^2^ or greater are considered overweight and obese [1]. The incidence of obesity has surged since 1980 and now affects a third of the world’s population [2]. According a global report by the World Health Organization in 2016, approximately 2 billion adults are overweight, with around half a billion people being obese [3]. Obesity usually results from excessive consumption of energy relative to physical and metabolic activity over a long period [4]. This excessive food intake leads to abnormal deposition of fat, which accumulates as adipose tissue, resulting in weight gain and augmented risk of developing many chronic diseases [5]. Most notably, type 2 diabetes, hypertension, dyslipidemia, cardiovascular diseases, Alzheimer disease, metabolic syndrome, non-alcoholic fatty liver disease, and cancer risk are often observed in individuals with obesity [6, 7].

Obesity and cancer development are strongly associated: obesity contributes to 40% of all cancers and 60% of endometrial, postmenopausal breast, and colorectal cancers (the latter being more common in women (9.6%) than men (4.7%)) [8]. Cytokine synthesis in the adipose tissue of individuals with obesity creates a chronic inflammatory state that strongly contributes to the onset of comorbidities linked to obesity and increased risk of cancer development [9].

Treatment approaches include lifestyle changes (dieting and physical activity), cognitive behavioral therapy, pharmacotherapy, and surgery. Bariatric surgery results in sustainable and substantial weight loss and has proven to be more effective than other treatments [10]. Moreover, bariatric surgery may have a positive impact on altered glucose metabolism and reduce morbidity and mortality attributable to cancer and cardiovascular disease [11]. The potential adverse effects of bariatric surgery include recurrent calcium oxalate urolithiasis and osteoporosis [12]. However, there are few studies with long-term follow-up after bariatric procedures.

While genetic factors play an important role in determining individual susceptibility to weight gain and obesity [13], they do not account completely for the obese phenotype. The recent identification of distinct epigenetic patterns in individuals who are obese or have a metabolic imbalance suggests a potential role for epigenetic alterations in causal or mediating pathways in the development of obesity-related comorbidities and some types of cancer and cancer recurrence. Studies in animals and humans have shown that obese and metabolically unbalanced individuals have distinct epigenetic patterns that are reversible upon weight loss [14–16]. This has led to growing interest in understanding the potential role of epigenetic changes as mediators of gene–environment interactions underlying the development of obesity and associated comorbidities [17]. Epigenome alterations may play a causative role in obesity and its complications (including cancer) by inducing changes in gene expression [17]. Alternatively, but not mutually exclusively, epigenome deregulation may develop as a consequence of obesity and adiposopathy (i.e., altered pattern of adipokine secretions and related local and systemic low-grade chronic inflammation), and once established, could mediate their downstream effects (type 2 diabetes, cardiovascular disease, and cancer) by changing gene activity states (transcriptional program) [18]. Interestingly, recent DNA methylome studies have suggested that unique epigenetic changes associated with obesity can be detected in the blood and that these changes are potentially reversible after weight loss [19].

One significant epigenetic modulator is DNA methylation, which is readily altered by environmental and lifestyle factors [20, 21]. Recent studies found DNA methylation-mediated epigenetic alterations at different time points after bariatric surgery in morbidly obese individuals [19, 22]. The reversible nature of DNA methylation has led to a hunt for target epigenetic markers for the treatment of obesity and related diseases [23]. Epigenetic alterations attributable to obesity might further influence complex life events including aging [21]. Generally, global hypomethylation and site-specific hypermethylation are common age-dependent alterations in DNA methylation [24]. Obesity is also intricately linked with aging and related comorbidities that shorten the life span and speed up aging [25]; studies report that higher BMI is associated with accelerated aging even in younger individuals [26]. These complex molecular interactions can be investigated via understanding weight loss-associated epigenetic alterations and related genes and pathways.

In the present study, we investigated the effect of bariatric surgery-induced weight loss on clinical parameters and epigenetic alterations in individuals with severe obesity. We collected blood samples and follow-up data and performed DNA methylome analysis to identify differentially methylated genes and pathways linked to weight loss. To substantiate our results, we included a replication set of samples from BMI-discordant monozygotic twins. The obese twins in the replication set lost weight due to caloric restriction, thus serving as a control group that did not undergo bariatric surgery. We also explored the association between weight loss and aging through the epigenetic clock [27]. The weight loss-associated epigenetic alterations we identified may be promising tools for diagnosing and understanding obesity and related diseases and for devising effective weight-loss therapies.

## Results

### Improvement in clinical and metabolic features after bariatric surgery

**Fig. 1:**
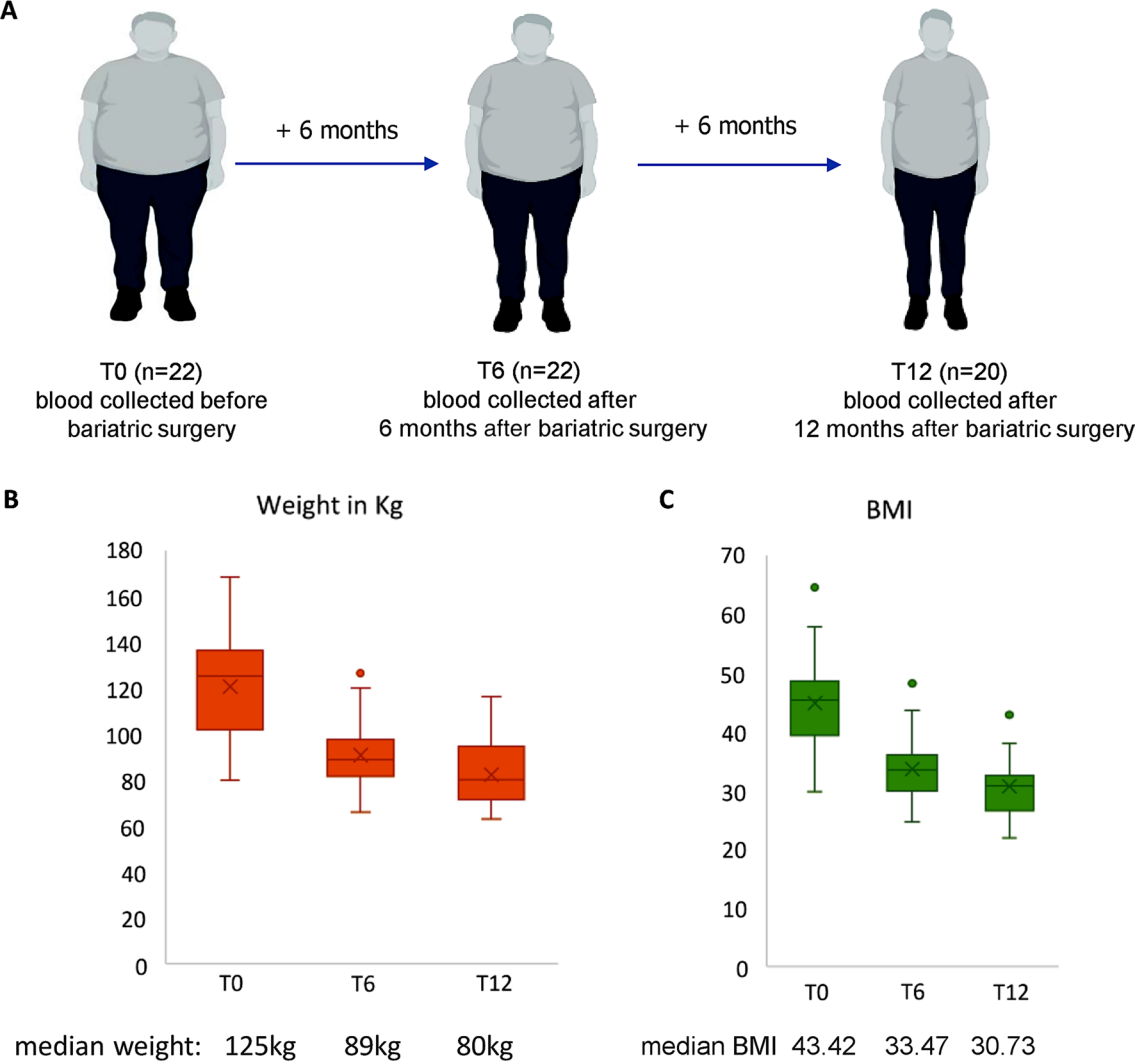
Study design **A** Subject details and collection timepoints; **B** Weight-loss trajectory at 6 and 12 months after bariatric surgery; **C** BMI-change trajectory at 6 and 12 months after bariatric surgery

We collected blood samples and clinical data from 22 subjects with obesity before they underwent bariatric surgery, and during two follow-up visits at 6 and 12 months after surgery (Fig. 1A). Most subjects were female (~ 91%) and the median age was 47.61 years (range 24–64 years). Detailed characteristics of the study population are shown in Table 1. Median weight at baseline was 125.3 kg (SD 23.86) and median BMI was 43.42 (SD 8.15). Six months after surgery, the subjects lost weight, reaching a median weight of 89.05 kg (SD 16.52) and a median BMI of 33.47 (SD 5.66). In addition to weight loss, the subjects also showed decreased levels of Homeostatic Model Assessment for Insulin Resistance (HOMA-IR; median 1.59 (SD 0.79) and hemoglobin A1c (HbA1c; median 33.5, SD 5.42) indicating an improvement in glycemic control. At 12 months after surgery, the subjects lost more weight (median 80.15 kg, SD 15.16) and reduced their BMI (median 30.73, SD 5.1) (Fig. 1B, C), while levels of HOMA-IR further improved.

**Table 1:** Clinical characteristics of the study population before and after bariatric surgery

Discovery	T0 (Baseline)	T6 (6 months)	T12 (12 months)	*p*-Value T0 vs. T6	*p*-Value T6 vs. T12	*p*-Value T0 vs. T12
N subjects	22	22	20			
Male/Female (n)	2/20	2/20	2/18			
Age (years)	47.61 (41.93–55.75)	48.21 (42.53–56.35)	48.81 (43.13–56.95)			
Height	163.5 ( 157.8–167.3)	163 (158–168)	161 (158–167.3)			
Weight	125.3 (100.8–138.5)	89.05 (80.45–99.43)	80.15 (69.15–94.75)	< 0.001	< 0.001	< 0.001
BMI (kg/m2)	43.42 (39.64–50.06)	33.47 (29.88–36.04)	30.73 (26.53–32.50)	< 0.001	< 0.001	< 0.001
Waist circumference (cm)	122 (115–130)	101 (93.75–110.8)	95 (85.25–106.8)	< 0.001	< 0.001	< 0.001
Hip circumference (cm)	142 (128–150)	123.3 (111.5–127.3)	115 (103.5–124.5)	< 0.001	< 0.001	0.0057
HOMA-IR*	4.32 (3.15–7.81)	1.59 (1.16–2.57)	1.54 (1.03–2.02)	< 0.001	0.3118	< 0.001
HbA1c (mmol/mol)	39 (36–44)	33.5 (31.75–35.5)	34.5 (32–36.75)	< 0.001	0.6154	< 0.001
Hypertension (n)	6 (27.27%)	3 (13.63%)	3 (15%)			
Dyslipidemia (n)	15 (68.18%)	10 (45.45%)	7 (35%)			
Impaired fasting glucose (n)	6 (27.27%)	1 (4.54%)	1 (5%)			

Continuous variables are expressed as median (IQ range) and discrete variables are reported as the number of subjects (%). Wilcoxon signed-rank test was used to analyze intergroup differences before surgery and 6 and 12 months after bariatric surgery

^*^HOMA-IR = Homeostatic Model Assessment for Insulin Resistance

### Enrichment of weight loss-associated DMPs in immune and inflammatory signaling pathways

**Fig. 2:**
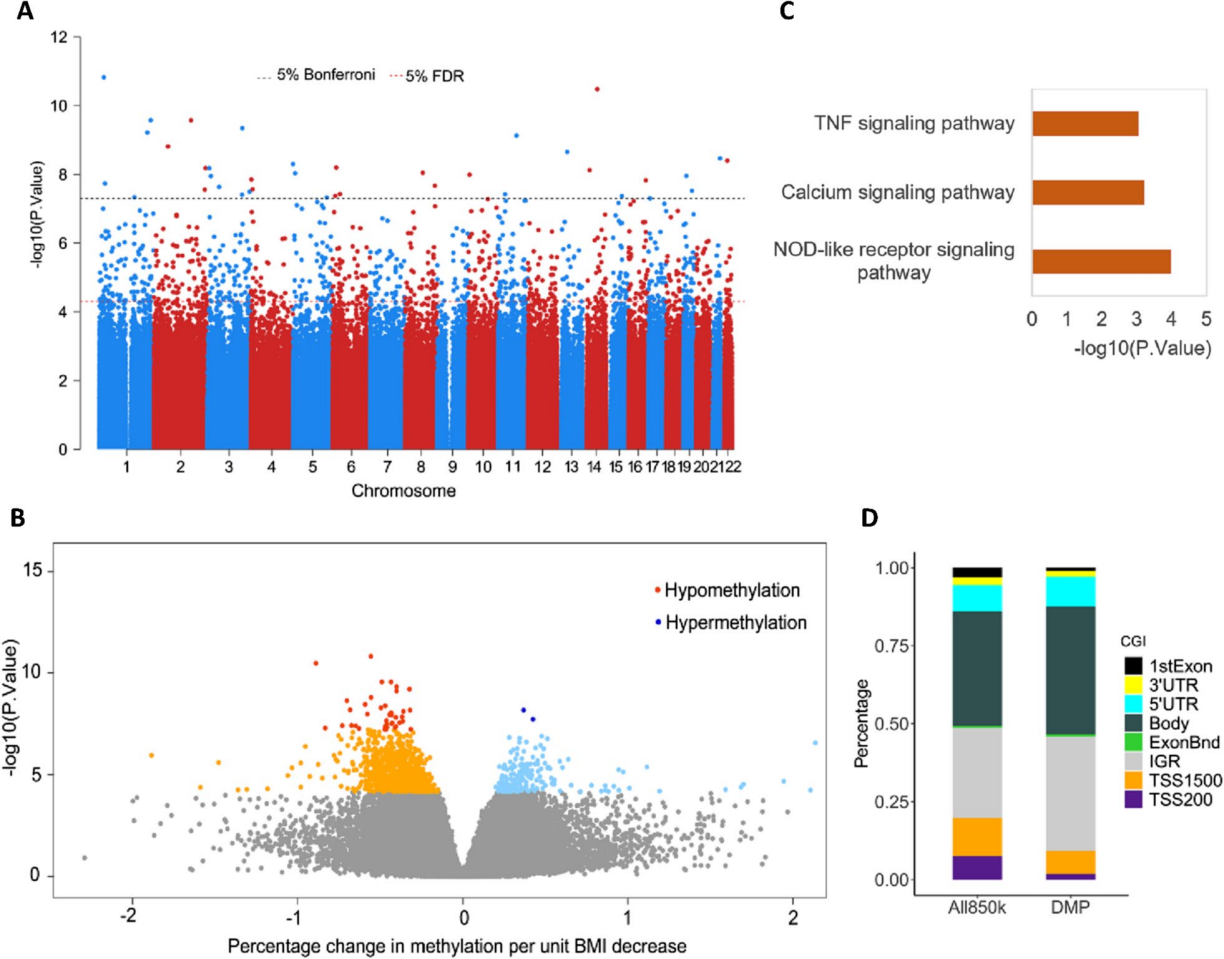
DMP analysis results **A** Manhattan plot showing all DMPs across autosomes; **B** Volcano plot showing hypermethylated and hypomethylated DMPs; **C** Significant pathway enrichment results using the identified DMPs; **D** Genomic localization of the identified DMPs

Epigenome-wide association analysis revealed 41 significant (Bonferroni *p* < 0.05) and 1169 suggestive differentially methylated CpGs (DMP, false discovery rate FDR *p* < 0.05) associated with weight loss due to bariatric surgery (Fig. 2A) (Additional file 2: Table T2). Most of the CpGs were hypomethylated (975/1169), i.e., DMPs exhibited decreased methylation levels (mean 0.40%, range 0.15–1.88% decrease in methylation for 1 unit decrease in BMI) with weight loss (Fig. 2B) and were spread across all chromosomes and enriched in TNF signaling, calcium signaling, and NOD-like receptor signaling pathways (Fig. 2C). Plasma secretome analysis of several inflammation mediators consistently showed a chronic low-grade inflammatory profile at baseline. A significant reduction in these markers was observed after bariatric surgery (related results being published in another article).

**Fig. 3:**
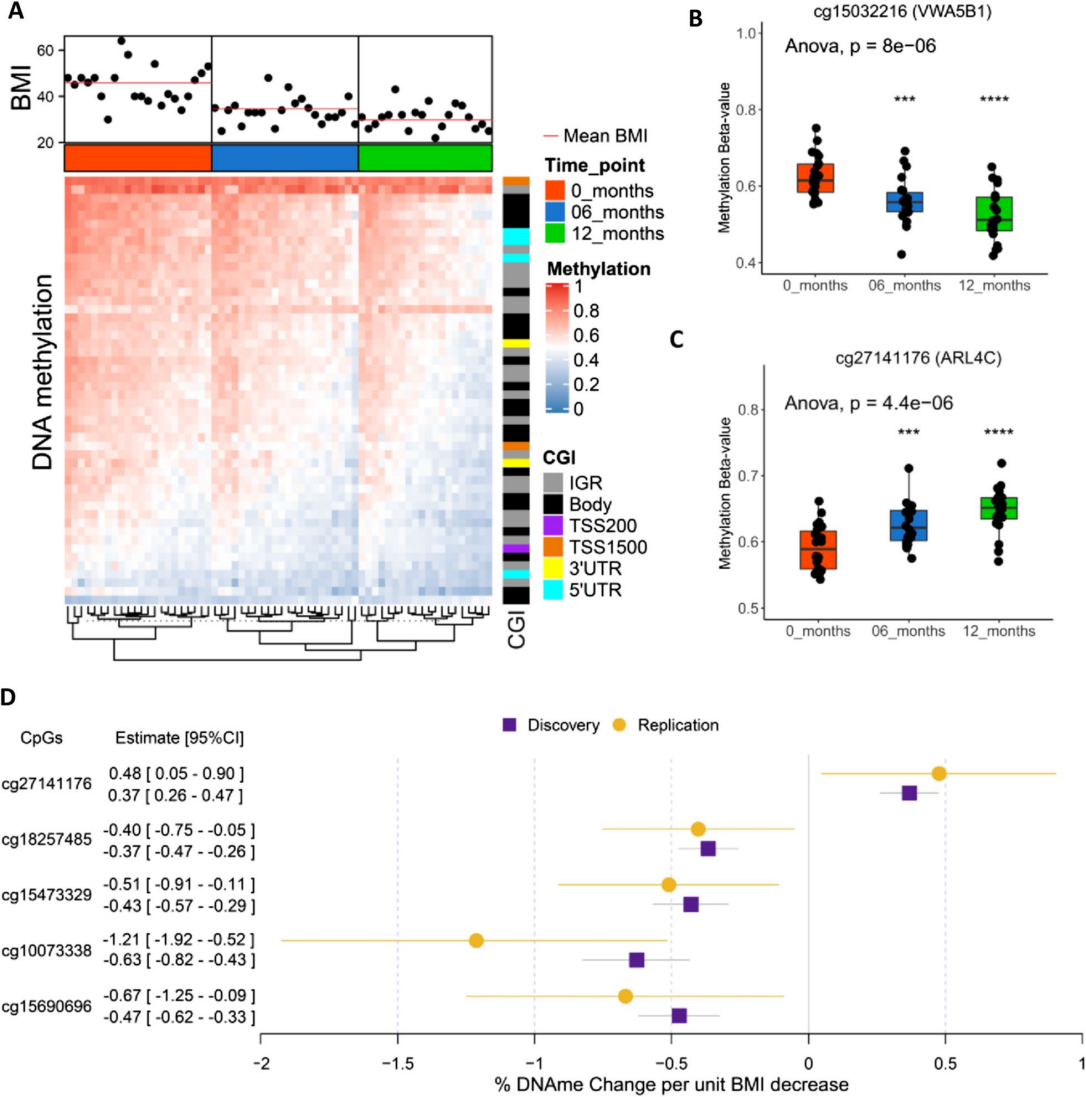
DMP characteristics and replication **A** Heat-map showing DNA-methylation patterns of DMPs with weight loss; **B** Top hypomethylated and **C** Hypermethylated DMP probe; **D** Forest plot showing DNA-methylation change per unit BMI decrease in discovery and replication set. (*UTR* Untranslated region; *IGR* Intragenic regions; *TSS* Transcriptional start site)

The DMPs were enriched in the intragenic regions, gene-body, and 5’UTR regions of the genome (Fig. 2D). The 41 significant CpGs included 39 hypomethylated and two hypermethylated CpGs (Table 1). Supervised clustering of the 41 CpGs showed clear hypomethylation associated with weight loss over 6-month intervals from 0 to 12 months (Fig. 3A and Table 2). Top hypomethylated CpG (cg15032216) mapped to the gene encoding Von Willebrand Factor A Domain Containing protein 5B1 (*VWA5B1*) on chromosome 1 (methylation change: − 0.56% [95%CI: − 0.68% to − 0.43%], *p* = 1.51 × 10^−11^) (Fig. 3B). Top hypermethylated CpG (cg27141176) mapped to the intergenic region of ADP Ribosylation Factor Like GTPase 4C (*ARL4C*) (methylation change: 0.37% [0.26% to 0.47%], *p* = 6.57 × 10^−9^) (Fig. 3C). Both genes, *VWA5B1* and *ARL4C*, have been previously reported as differentially methylated in obese subjects with related comorbidities [28, 29].

**Table 2:** A detailed description of Bonferroni-corrected 41 DMPs

CpG ID	Chromosome	Position	Gene	Estimate [95% CI]	*p*-value	Bonferroni
cg15032216	chr1	20,577,301	*VWA5B1*	−0.56 [−0.68 to −0.43]	1.51E-11	1.28E-05
cg12696263	chr14	67,446,489	*ATP6V1D*	−0.89 [−1.11 to −0.68]	3.32E-11	2.82E-05
cg05485304	chr1	234,382,487	*TARBP1*	−0.49 [−0.61 to −0.37]	2.67E-10	2.26E-04
cg17280849	chr2	168,783,262	*B3GALT1*	−0.43 [−0.55 to −0.32]	2.68E-10	2.27E-04
cg04504681	chr3	159,604,035	*IQCJ-SCHIP1*	−0.40 [−0.50 to −0.30]	4.54E-10	3.85E-04
cg03988297	chr1	219,060,547	*LYPLAL1*	−0.32 [−0.41 to −0.24]	6.07E-10	5.15E-04
cg11846494	chr11	83,393,148	*DLG2*	−0.40 [−0.51 to −0.29]	7.45E-10	6.33E-04
cg05424884	chr2	63,882,416	*WDPCP*	−0.55 [−0.71 to −0.40]	1.55E-09	1.31E-03
cg21957311	chr13	45,623,331	*NUFIP1*	−0.70 [−0.89 to −0.51]	2.23E-09	1.89E-03
cg24130641	chr21	45,478,471	*ICOSLG*	−0.59 [−0.76 to −0.42]	3.42E-09	2.90E-03
cg00334274	chr22	30,474,915	*HORMAD2*	−0.47 [−0.60 to −0.34]	4.00E-09	3.39E-03
cg05934812	chr5	334,322	*AHRR*	−0.49 [−0.64 to −0.35]	5.02E-09	4.26E-03
cg14928941	chr6	16,420,347	*ATXN1*	−0.68 [−0.88 to −0.48]	6.35E-09	5.38E-03
cg27141176	chr2	235,272,562	*ARL4C*	0.37 [ 0.26 to 0.47]	6.57E-09	5.57E-03
cg07904045	chr3	8,782,064	*SSUH2*	−0.32 [−0.41 to −0.23]	6.60E-09	5.60E-03
cg08144425	chr14	32,700,524	*ARHGAP5*	−0.36 [−0.46 to −0.25]	7.51E-09	6.37E-03
cg10119914	chr8	80,656,048	*STMN2*	−0.43 [−0.56 to −0.31]	8.98E-09	7.62E-03
cg27033979	chr5	10,030,097	*CCT5*	−0.43 [−0.56 to −0.30]	9.29E-09	7.89E-03
cg19726711	chr10	6,183,872	*MIR3155B*	−0.58 [−0.75 to −0.41]	1.03E-08	8.70E-03
cg14753070	chr19	14,163,842	*PALM3*	−0.44 [−0.57 to −0.31]	1.11E-08	9.42E-03
cg04978197	chr3	16,164,375	*DPH3*	−0.43 [−0.56 to −0.30]	1.12E-08	9.54E-03
cg18257485	chr4	2,956,627	*NOP14*	−0.37 [−0.47 to −0.26]	1.41E-08	1.20E-02
cg00656990	chr16	78,812,407	*WWOX*	−0.40 [−0.53 to −0.28]	1.49E-08	1.26E-02
cg06092310	chr1	26,097,918	*MAN1C1*	0.42 [ 0.30 to 0.55]	1.85E-08	1.57E-02
cg06312004	chr8	135,836,721	*MIR30D*	−0.46 [−0.60 to −0.32]	2.15E-08	1.82E-02
cg15518106	chr3	53,836,983	*CHDH*	−0.36 [−0.48 to −0.25]	2.32E-08	1.97E-02
cg09074744	chr4	8,601,496	*CPZ*	−0.38 [−0.50 to −0.27]	2.73E-08	2.32E-02
cg16472834	chr2	231,533,021	*CAB39*	−0.43 [−0.56 to −0.30]	2.79E-08	2.37E-02
cg15473329	chr19	39,862,918	*SAMD4B*	−0.43 [−0.57 to −0.29]	3.01E-08	2.55E-02
cg23282115	chr3	192,711,447	*MB21D2*	−0.46 [−0.60 to −0.32]	3.20E-08	2.72E-02
cg05352838	chr6	33,384,391	*CUTA*	−0.67 [−0.89 to −0.46]	3.75E-08	3.18E-02
cg00797434	chr11	32,680,447	*EIF3M*	−0.73 [−0.96 to −0.50]	3.80E-08	3.23E-02
cg07255810	chr3	158,161,558	*LXN*	−0.65 [−0.84 to −0.45]	3.93E-08	3.33E-02
cg19356286	chr6	11,722,624	*TMEM170B*	−0.40 [−0.53 to −0.28]	4.25E-08	3.60E-02
cg00470050	chr15	72,206,554	*MYO9A*	−0.47 [−0.62 to −0.32]	4.28E-08	3.63E-02
cg21022868	chr1	159,923,861	*LINC01133*	−0.46 [−0.60 to −0.32]	4.65E-08	3.94E-02
cg05945665	chr5	153,986,184	*LARP1*	−0.41 [−0.54 to −0.28]	4.71E-08	4.00E-02
cg13407335	chr17	7,832,852	*KCNAB3*	−0.83 [−1.10 to −0.57]	5.00E-08	4.24E-02
cg10073338	chr10	89,521,994	*ATAD1*	−0.63 [−0.82 to −0.43]	5.26E-08	4.46E-02
cg23868054	chr11	124,764,510	*ROBO4*	−0.31 [−0.41 to −0.21]	5.76E-08	4.89E-02
cg15690696	chr11	120,217,718	*ARHGEF12*	−0.47 [−0.62 to −0.33]	5.81E-08	4.93E-02

To examine whether the association between BMI and methylation is influenced by time, we performed an additional linear mixed model (LMM) with an interaction term between BMI and time as fixed effects. The analysis showed that the interaction did not reach genome-wide statistical significance for any of the CpGs tested (Additional file 1: Fig. S1). For the significant CpGs, there was no evidence of any significant interaction effect (nominal *p* > 0.05) between BMI and time. Therefore, our results are strongly associated with BMI change and not influenced by time.

### Weight loss-associated DMPs common to bariatric surgery and hypocaloric dieting

The 41 differentially methylated CpGs (Bonferroni *p* < 0.05) were used to test the results in an independent replication set consisting of peripheral blood mononuclear cells (PBMCs) from eight healthy pairs of monozygotic BMI-discordant twins undergoing a hypocaloric diet. As expected, a low-calorie diet induced fewer epigenetic changes than did bariatric surgery, in line with the modest BMI change associated with dieting (< 10%); however, 5 CpGs were replicated at *p* < 0.05 in the replication set. The effect sizes were consistent across the discovery and replication sets, meaning that the percentage β value change per unit BMI decrease was similar among the 5 CpGs in both the sample sets (Fig. 3D). Of these 5 CpGs, cg27141176 mapped to the *ARL4C* gene, which was hypermethylated upon weight loss, with a methylation change of 0.37% in the discovery vs 0.48% in the replication set per unit decrease in BMI. Of the replicated hypomethylated CpGs, cg10073338 (*ATAD1*), cg15473329 (*SAMD4B*), cg18257485 (*NOP14*), and cg15690696 (*ARHGEF12*) exhibited methylation changes of − 0.63%, − 0.43%, − 0.37% and − 0.47% in the discovery set vs − 1.21%, − 0.51%, − 0.40% and − 0.67% in the replication set, respectively, per unit change in BMI (Fig. 3D). These CpGs all map in genes acting as molecular switches in different metabolic pathways (https://www.genecards.org/). Due to the small sample size in the replication set, we also compared the effect sizes of association in the discovery and replication sets and observed a significant correlation (*p* = 0.0006, Additional file 1: Fig. S2).

### Weight loss-specific DMRs identify genes involved in tissue homeostasis

**Fig. 4:**
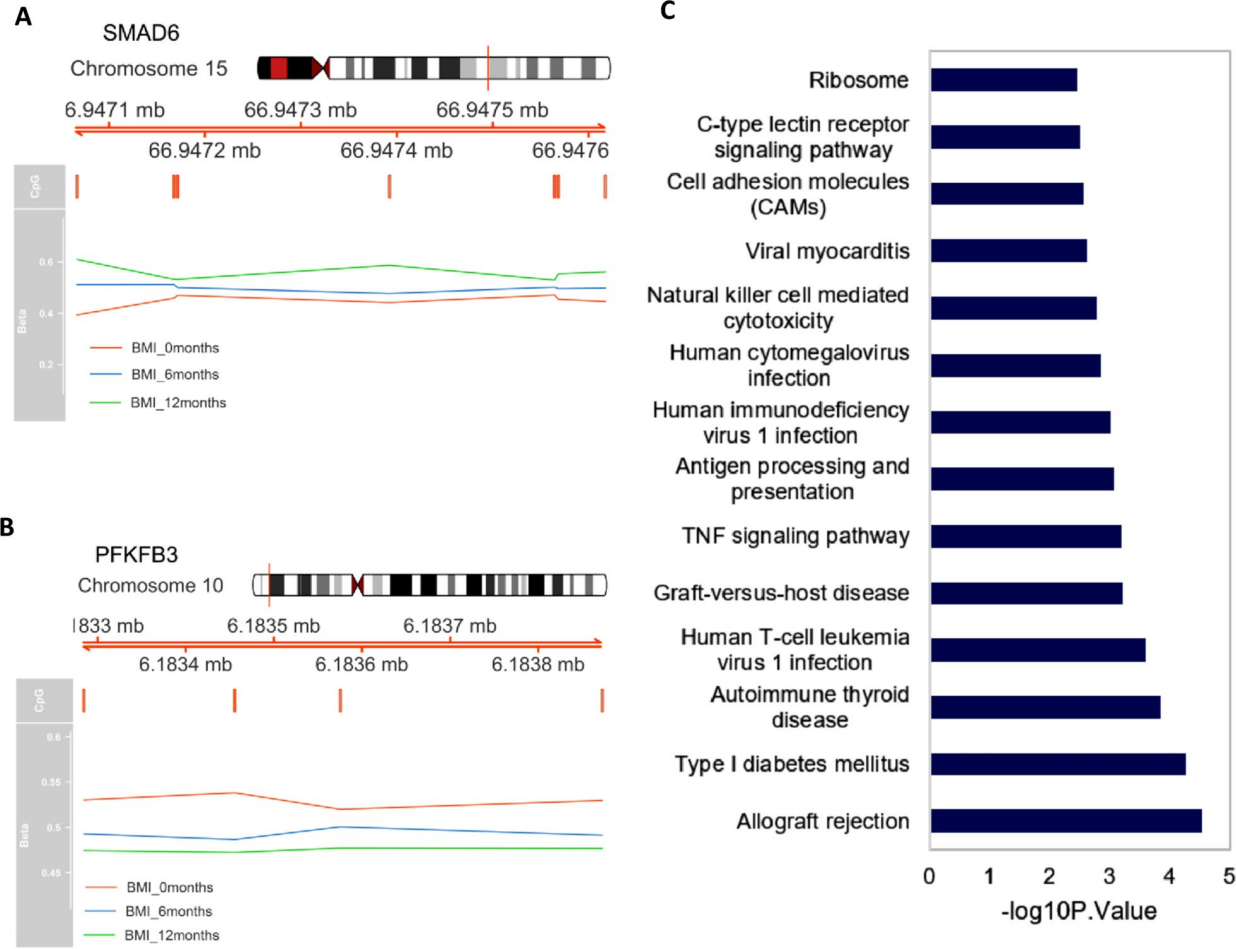
DMR plots and associated pathways **A** Top hypermethylated region/nearest gene SMAD6 and **B** top hypomethylated gene PFKFB3, **C** Pathway enrichment analysis of DMR associated genes

Methylome-wide analysis before and after weight loss identified 192 DMRs with at least 2 CpGs within the 500-bp window and FDR < 0.05. Of these DMRs, 120/192 (62.5%) were hypomethylated and 72/192 (37.5%) were hypermethylated (Additional file 2: Table T3). *SMAD6* was the nearest gene located downstream to the top hypermethylated DMRs and the top hypomethylated DMR was in the promoter region of the *PFKFB3* gene (Fig. 4A, B). SMAD6 and PFKFB3 are key regulators of tissue homeostasis (https://www.genecards.org/). Both these top DMRs overlap with sites of histone H3K27 acetylation, which is a marker of active chromatin (Additional file 1: Fig. S3). The H3K27 acetylation sites at the promoter region of the *PFKFB3* gene are clear, suggesting that aberrant methylation in this DMR might influence the expression of the *PFKFB3* gene. Pathway enrichment analysis of the DMR-associated genes showed that functional pathways related to the immune system and type 1 diabetes were significantly enriched (Fig. 4C).

### Weight loss decelerates epigenetic aging

**Fig. 5:**
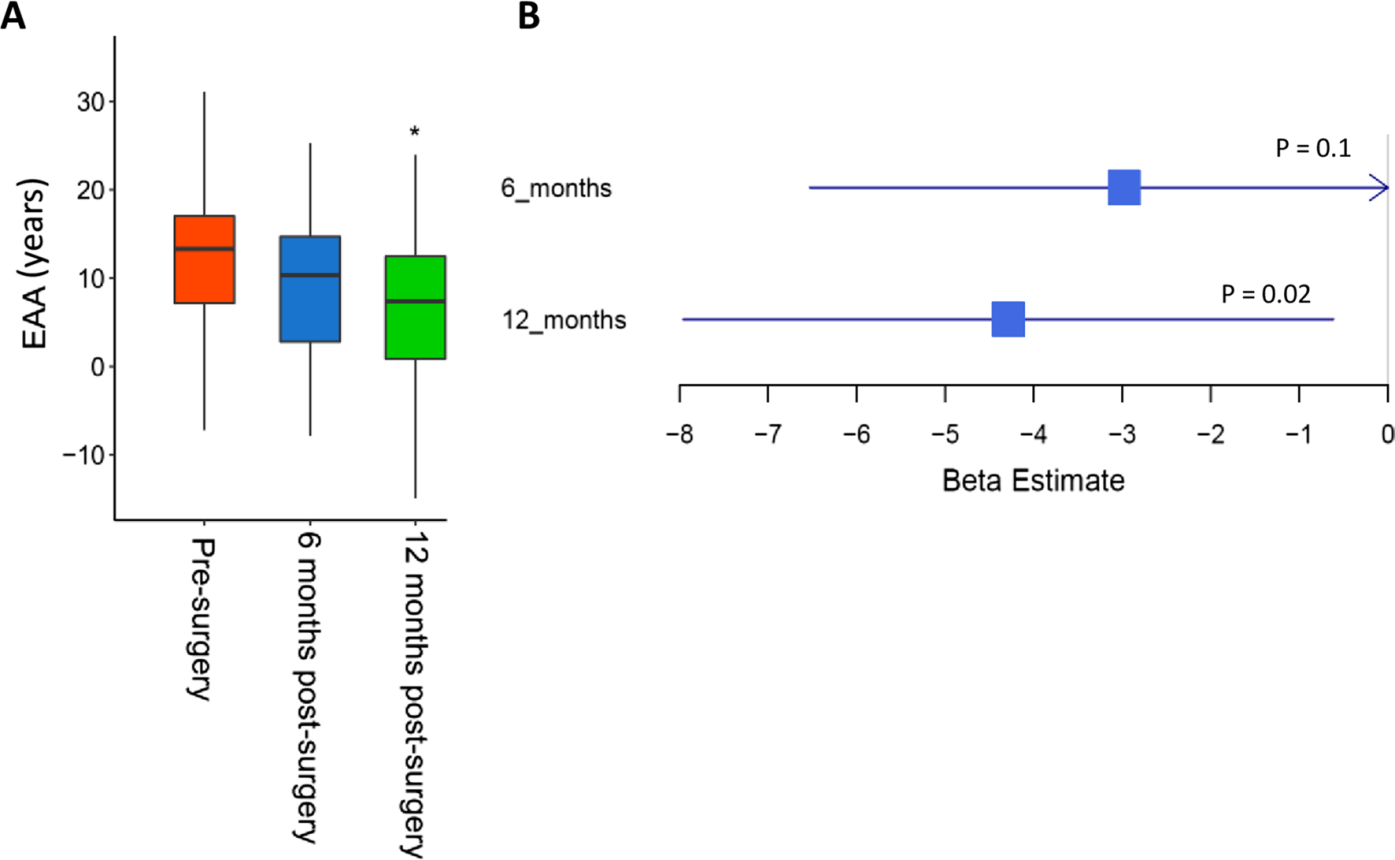
Epigenetic age acceleration (EAA) analysis using the Hannum et.al. method. It is determined by taking the residual from the regression of epigenetic age (based on β values of 71 CpG probes) on chronological age. Positive EAA values thus suggest that the epigenetic age is greater than expected based on chronological age. **A** EAA at different time points through the course of weight loss. **B** Regression estimates (beta estimate) were analyzed considering the time point 0 (pre-surgery) as reference

Because the acceleration of the “epigenetic clock” (a highly accurate multi-tissue biomarker of aging) has been associated with susceptibility to various pathological conditions [31, 32] including obesity [33, 34], we investigated the impact of weight loss on epigenetic age acceleration (EAA) by comparing epigenetic age before and after surgery [29]. A trend toward decelerated epigenetic aging was observed from pre-surgery to 6-month post-surgery. The association between decelerated epigenetic aging and weight loss reached significance 12-month post-surgery (EAA estimate − 4.29 (CI, − 7.95 to − 0.62), *p* = 0.02) (Fig. 5A). Our findings provide further evidence that bariatric surgery brings about an improvement in biological age and in the clinical/metabolic profile of the patient (Fig. 5B) and support the concept that obesity and normal ageing share numerous biological similarities [35, 36].

## Discussion

For people with severe obesity, bariatric surgery is becoming one of the most effective clinical interventions to achieve considerable and sustained weight loss together with restoration of metabolic homeostasis and overall health profile [37], although detrimental outcomes associated with metabolic surgery have also been reported [38].

In this study of the clinical and metabolic effects of bariatric surgery in patients with severe obesity (mean BMI ~ 45), we analyzed epigenetic alterations and molecular aging in PBMCs collected before and 6 and 12 months after surgery. The main advantage of our study was that we collected samples at three different time points, minimizing the effects of other confounding variables, and we note that the sample size was limited and the timing of follow up did not take into account long-term effects after surgery, such as potential weight regain after 12 months. Blood was selected as the target tissue, being easily accessible and a useful tool for the performance of longitudinal epigenetic studies to identify obesity-related biomarkers for intervention strategies. Our methylome analysis was performed with Illumina Methylation EPIC arrays that interrogate more than 850,000 CpGs across the genome, with almost double the coverage of previous studies [22].

### Alterations in DNA methylation are associated with metabolic health

In the study population, restoration of metabolic health, as indicated by improvement in glycemic control, hypertension, and dyslipidemia, was observed in association with rapid and significant reduction (30% reduction) in body weight, mostly occurring within 6 months after surgery. DNA methylome analysis of PBMCs isolated before and after surgery identified 41 (Bonferroni corrected *p*-value) weight loss-specific differentially methylated positions (DMPs) across autosomes, preferentially located in the intergenic regions (IGRs). Gradual hypomethylation was observed across the differentially methylated CpGs in association with the restoration of clinical and metabolic health. Many other studies have also reported that methylation level, mostly hypomethylation, at CpG sites changes with BMI value [16, 39] and is associated with a healthier metabolic status. Our findings add to the evidence that bariatric surgery can restore DNA-methylation profiles in patients with obesity to resemble profiles seen in healthy people [22]. The challenging question remains whether there is a cause–effect relationship between weight loss and DNA methylation [40]. Changes in DNA methylation may lead to deregulation of critical cell signaling pathways [41]. In our study, pathway analysis showed that the genes associated with weight-loss-specific DMPs were enriched in pathways involving TNF, NOD-like receptors, and calcium signaling.

The TNF-signaling pathway is a critical crosslink between obesity and metaflammation. Altered TNF signaling pathways promote hyperglycemia, hyperlipidemia, and low-grade chronic inflammation, increasing the risk of developing non-communicable metabolic diseases such as type 2 diabetes, non-alcoholic fatty liver disease, cardiovascular disease, chronic kidney disease, and airway disease [42]. In obese people, this pathway mainly participates in oxidative stress, inflammation, and angiogenesis, increasing risk of cancer development [43].

NOD-like receptor signaling is active in many chronic diseases, including obesity. NOD-like receptor protein 3 (NLRP3) is a well-characterized inflammasome responsible for inflammation and insulin-resistance development [44, 45] and is also associated with atrial fibrillation (another obesity-related comorbidity) [46, 47]. Although the evidence regarding the effects of bariatric surgery on DNA methylation in humans is limited and often inconsistent [39], other studies indicate that bariatric surgery modulates the methylation of genes belonging to inflammatory pathways [30, 48], leading to changes in the circulating levels of inflammatory markers [49].

The calcium-signaling pathway is also associated with the progression of obesity and many other diseases. Calcium ions can impede obesity development by facilitating the differentiation of adipocytes and metabolism to enhance energy consumption [50, 51]. Calcium signaling could be involved in the neural control of hunger and energy metabolism as well as in the gut–brain axis [52, 53]. In line with our study, another genome-wide DNA methylome study conducted on people with obesity reported calcium-signaling among the top five enriched pathways [54].

### Energy restriction and bariatric surgery present common CpG sites

Another open question in the field is whether the weight-loss strategy selected, in particular, energy restriction vs bariatric surgery, has a differential effect on the DNA-methylation profile and which genes are involved. To address this question, we used a small cohort of monozygotic twins who were discordant for BMI as replication for our discovery set of Bonferroni-corrected CpGs. The obese twin lost weight by caloric restriction, and the inclusion of the corresponding healthy lean twin in the analysis nullified the effect of time. In addition to confirming that bariatric surgery has a broader impact on the methylation profile than does a hypocaloric diet [30], we identified 5 CpGs that were replicated in the discovery and replication set. DNA-methylation changes per unit decrease in BMI were consistent in the discovery and replication samples, suggesting that the identified markers were exclusively associated with weight loss itself and not with the weight-loss procedure. These CpGs all mapped to genes encoding molecular regulators in various signaling pathways, including GTP-binding protein ARL4C, mitochondrial translocase ATAD1, transcription factor SAMD4B, RNA-binding protein NOP14, and RhoA GTPase regulatory protein ARHGEF12 (https://www.genecards.org/).

These findings indicate that specific epigenetic changes occur as early events in response to weight loss, even when the loss does not exceed 10% of the initial body weight (as was the case for the overweight twin undergoing hypocaloric dieting). The weight loss-specific significant hypermethylation of *ARL4C* identified in both the discovery and the replication set deserves a comment. Recent studies show that expression of *ARL4C* induces proliferation in endometrial cancer cells; expression is significantly decreased after exposure to metformin (the most common anti-diabetic drug), leading to reduced proliferation [55]. Recent studies have shown that the use of metformin in obese patients with endometrial cancer prolongs life expectancy and reduces the risk of recurrence [56, 57]. Overall, these data suggest that *ARL4C* methylation may be a promising obesity-related epigenetic marker.

### Epigenetic regulation is key in the pathophysiology of obesity

In this study, we identified weight loss-specific DMRs associated with bariatric surgery. Pathway enrichment analysis showed modulation of pathways involved in the immune system and in type 1 diabetes. The first two closest DMR genes identified were *SMAD6* and *PFKFB3*, which have pleiotropic functions in the maintenance of tissue homeostasis. Both genes play a role in endothelial and adipose tissue dysfunction in obesity. Several studies indicate that aberrant expression of *SMAD6*, a negative regulator of TGF-α signaling, affects homeostasis of the cardiovascular system [58, 59] and plays a role in obesity in a mouse model [60]**.** The *PFKFB3* gene, which encodes a key glycolytic activator, also plays a crucial role in maintaining endothelial function and, when defective, reduces pathological angiogenesis in mice [61]. On the basis of these findings, we speculate that the modulation of methylation levels of *SMAD6* and *PFKFB3* after surgery observed in our study might be the underlying mechanism for the reduction in cardiovascular risk associated with bariatric surgery [62].

In addition, both genes are implicated in adipose tissue dysfunction, which characterizes severe obesity. TGF-α/SMAD6 signaling is implicated in the white adipocyte commitment of mesenchymal stem cells [63] and levels of expression of *PFKB3* in adipose tissue affect both inflammation and insulin sensitivity [64, 65]. Although the current evidence suggests that epigenetic modifications of these genes might be potential markers of obesity and associated diseases, further studies are required to define their functional relevance.

## Conclusions

Our results confirm that bariatric surgery-induced weight loss results in an improvement in clinical and metabolic parameters and leads to epigenetic changes in response to the establishment of a new equilibrium. The changes in methylome pattern and clinical profiles observed after weight loss strongly suggest that epigenetics might play a crucial role in the health improvements observed in our cohort. We also replicated the top targets identified from the discovery set in a small group of BMI-discordant monozygotic twins who followed a hypocaloric diet. Partial overlap of 5 DMPs was observed, suggesting that these particular changes are due to weight loss per se regardless of how it was achieved. Our findings confirm the association between bariatric surgery-induced methylation changes and decelerated epigenetic aging, thus reinforcing the concept that obesity has numerous biological similarities to the normal aging process.

Further studies are needed to investigate the role of these epigenetic changes at the functional level to gain insight into a potential physiopathological role in obesity. Our results could aid the identification and monitoring of DNA methylation-based biomarkers of obesity and associated complications for therapeutic and diagnostic usage in the future.

## Methods

### Sample collection and study design

A discovery cohort of 22 severely obese patients undergoing bariatric surgery, either sleeve gastrectomy (19/22, 86%) or Roux-en-Y gastric bypass (3/22 (4%), was enrolled from February 2017 to March 2019 at the Obesity Center of University Hospital Policlinico Tor Vergata, (Rome, Italy). All surgical procedures were performed laparoscopically. All patients underwent a comprehensive medical and anthropometric evaluation, as well as a body composition analysis. Blood samples were collected and PBMCs were isolated at baseline (pre-surgery, T0) and 6 months (T6) and 12 months (T12) after sleeve gastrectomy and processed. The study design and follow-up procedures are described in Fig. 1A. The inclusion criteria were as follows: 18 > age < 60; 35 > BMI < 50 (kg/m^2^); individuals with hypertension and liver steatosis were also included. Among the exclusion criteria were: patients with type 2 diabetes mellitus or taking hypoglycemic drugs; chronic liver or kidney disease, infections, malignancy, or other acute or chronic systemic or degenerative diseases; or use of glucocorticoids, non-steroidal anti-inflammatory medications, or antibiotics prebiotics, and probiotics in the month before enrollment; any intake of drugs with known major impact on the gastrointestinal tract in the 3 months before enrollment; and pregnant or lactating women. All patients signed informed consent for participation in the study and all relevant information on family and medical history, current drug consumption, dietary, smoking habits, physical activity, and psychological state were recorded by a validated detailed questionnaire. On the surgery day, an interview was carried out for the collection of anamnestic data, and anthropometric (weight, height, waist, and hip circumference) and clinical (blood pressure, heart rate) parameters were recorded. After surgery, patients received periodic counseling about long-term dietary modifications, focusing on the adaptation of eating behavior to the surgical procedure and the general qualitative aspects of a healthy nutrient-dense diet. Protein intake up to 1.5 g/kg ideal body weight per day and regular physical activity were encouraged and long-term mineral and multivitamin supplementation prescribed as recommended [66]. Patients were advised to practice moderate aerobic physical activity, from a minimum of 150 min/week (with a goal of 300 min/week) as well as strength training 2–3 times per week.

This study was approved by the ethical committees of Istituto Superiore di Sanità (prot.PRE 173,116 of 15 March 2016; PRE-BIO-CE 10,938 of 6 April 2018) and of University Hospital Policlinico Tor Vergata (protocol of the study connecting DNA 169/15) and a written informed consent was obtained from all subjects.

### Replication set

A cohort of 8 healthy pairs of twins discordant for BMI (8 normal weight and 8 overweight individuals) underwent a diet regimen or caloric restriction for a weight loss of 10% of the original weight in approximately 6 months. This cohort was used as a replication set and the clinical characteristics are shown in Additional file 2: Table T1. DNA methylation was quantified in PBMC samples collected before and after dieting and used as a replication series for the top findings of the discovery set.

### Sample processing

The Norgen genomic DNA isolation kit (Norgen Biotek Corp. catalogue No. 24700) was used to isolate total DNA from PBMC according to the manufacturer's instructions. DNA was quantified using a Qubit 3 Fluorometer with the Qubit dsDNA BR Assay Kit (Thermo Fisher Scientific) according to the manufacturer’s protocol. Around 500 ng of DNA, from each sample, was then processed for bisulfite conversion using the EZ-DNAMethylation Kit (Zymo Research) and hybridized using the Infinium Methylation EPIC Kit (San Diego, CA, USA) or HM850K as illustrated by the manufacturer [67, 68]. The Intensity Data (IDAT) files generated from the array were then used for downstream methylome analyses.

### DNA methylome analysis

The HM850K array can interrogate DNA-methylation status of > 850,000 CpG sites at single-base resolution. The raw IDAT files were pre-processed and normalized using the “minfi” Bioconductor package in R programming language with the R-Studio as described previously [69]. We excluded samples consisting of > 10% missing data for or overall low confidence for > 10% of CpG sites. Also, CpG sites showing low detection *p*-value (detection *p* > 0.05), probes associated with single-nucleotide polymorphisms [70], cross-reactive probes [71], and probes within X and Y chromosomes were removed from the analysis [68]. After the probe filtering, around 829,696 CpG sites remained for further analysis in which each CpG site was designated with a specific β value. The β value is determined by the ratio of signal intensities between methylated (M) and unmethylated (U) probes, which ranges from 0 to 1 (0 being unmethylated, and 1 for fully methylated). The “SVA” package was used to adjust potential known (batch, age, sex, etc.) and unknown confounders [72].

### Statistical analysis

To examine the effect of weight loss on the DNA methylome in PBMC after bariatric surgery, we used linear mixed models (LMM) with random intercepts and slopes for subjects to study the association between BMI and methylation over time. We used BMI as a proxy for weight to perform regression analysis with DNA methylation. The use of BMI considers the baseline differences in weight due to variation in height of the individuals. To test if the association between BMI and methylation is modified by time, we constructed additional LMM with an interaction term between BMI and time as fixed effects. Since multiple measurements were obtained per participant over time, LMM systematically accounts for within-subject variability while calculating the variation within groups over time. LMMs were constructed using the lme4 package and *p*-values obtained using the lmerTest package implemented in R [73]. For ease of interpretation, we used log-transformed methylation values as the dependent variable and expressed β coefficients from the regression analysis as the percentage change in methylation for a one-unit change in BMI. We used FDR corrected *p*-values to account for multiple testing. For replication, we used a more stringent Bonferroni corrected *p* < 0.05 in the discovery set to select differentially methylated positions (DMP) to be taken forward for replication. The differential methylated regions (DMR) were obtained using the DMRcate package by considering ≥ 2 CpGs within a 500-bp window that were differentially methylated due to weight loss.

### Pathway enrichment analysis

To identify functional pathways affected by weight loss, we performed pathway enrichment analysis using the online resource Enrichr [74]. For the analysis, only genes associated with DMP and DMR were included, and non-genomic regions were excluded. The Enrichr platform is a gene-set search engine that allows interrogation of a set of annotated genes and combines available knowledge to provide meaningful information about probably enriched pathways associated with the input gene sets. *p*-values for pathway enrichment were adjusted for multiple testing using a false discovery rate (FDR < 5%) to identify significantly enriched Kyoto Encyclopedia of Genes and Genomes pathways.

### Epigenetic age acceleration analysis

DNA methylome predicted age was calculated using the Hannum method as the sum of the beta values multiplied by the respective regression coefficients reported by Hannum et al. [27]. This provided a precise age estimate based on blood DNA-methylation levels at 71 CpG probes from the Illumina array [27]. EAA is determined by taking the residual from the regression of epigenetic age on chronological age. Positive EAA values thus suggest that epigenetic age is greater than expected based on chronological age. Here EAA was estimated by subtracting the chronological age from the predicted DNA-methylation age before and after surgery. The statistical significance of the change in EAA before and after surgery was determined using paired t tests and *p*-values < 0.05 were considered significant. We also used LMM to study the extent of decrease in EAA with time in response to weight loss.

## Supplementary Information

**Additional file 1: Figure S1.** Manhattan plot for EWAS, p-values of BMI x time interactions. **Figure S2.** Correlation of effect sizes of the 41 significant CpGs in discovery and replication sets. **Figure S3.** Genomic localization of the top DMR associated with SMAD6 and PFKFB3 genes.

**Additional file 2: Table T1.** Subject characteristics of replication set. **Table T2.** List od FDR (p<0.05) corrected DMP. **Table T3.** DMR list.

## Data Availability

The datasets used and/or analyzed during the current study are available from the corresponding author on reasonable request.
