# Supplementary material for: Bariatric surgery-induced weight loss and associated genome-wide DNA-methylation alterations in obese individuals
Source: Clin Epigenetics. 2022 Dec 18;14:176. doi: 10.1186/s13148-022-01401-9 (PMC9759858; doi:10.1186/s13148-022-01401-9)
Supplement: Supplementary file 1 — Additional file 1: Figure S1. Manhattan plot for EWAS, p-values of BMI x time interactions. Figure S2. Correlation of effect sizes of the 41 significant CpGs in discovery and replication sets. Figure S3. Genomic localization of the top DMR associated with SMAD6 and PFKFB3 genes. [file 13148_2022_1401_MOESM1_ESM.pptx]

## Slide 1
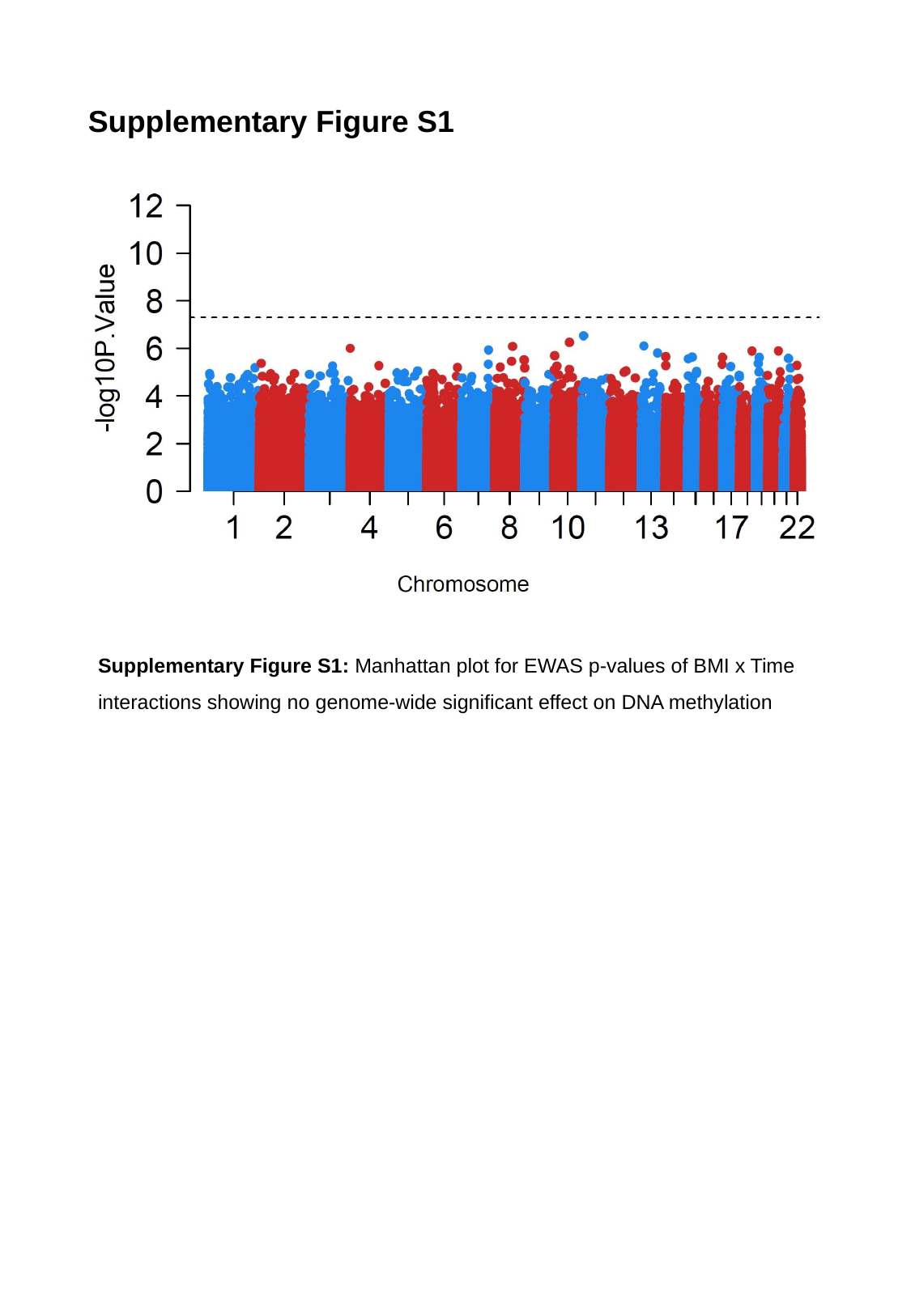

Supplementary Figure S1
Supplementary Figure S1: Manhattan plot for EWAS p-values of BMI x Time interactions showing no genome-wide significant effect on DNA methylation

## Slide 2
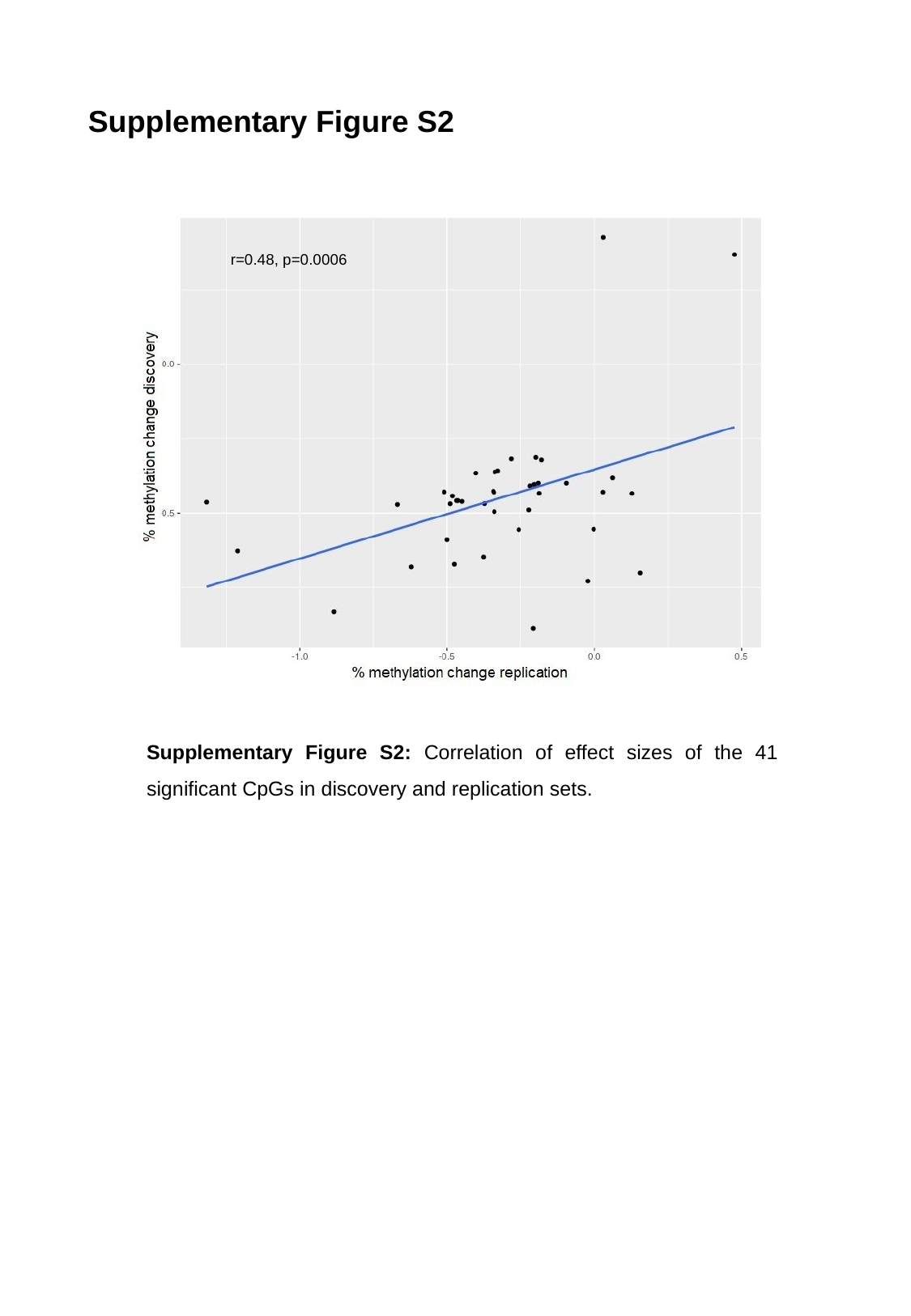

Supplementary Figure S2
r=0.48, p=0.0006
Supplementary Figure S2: Correlation of effect sizes of the 41 significant CpGs in discovery and replication sets.

## Slide 3
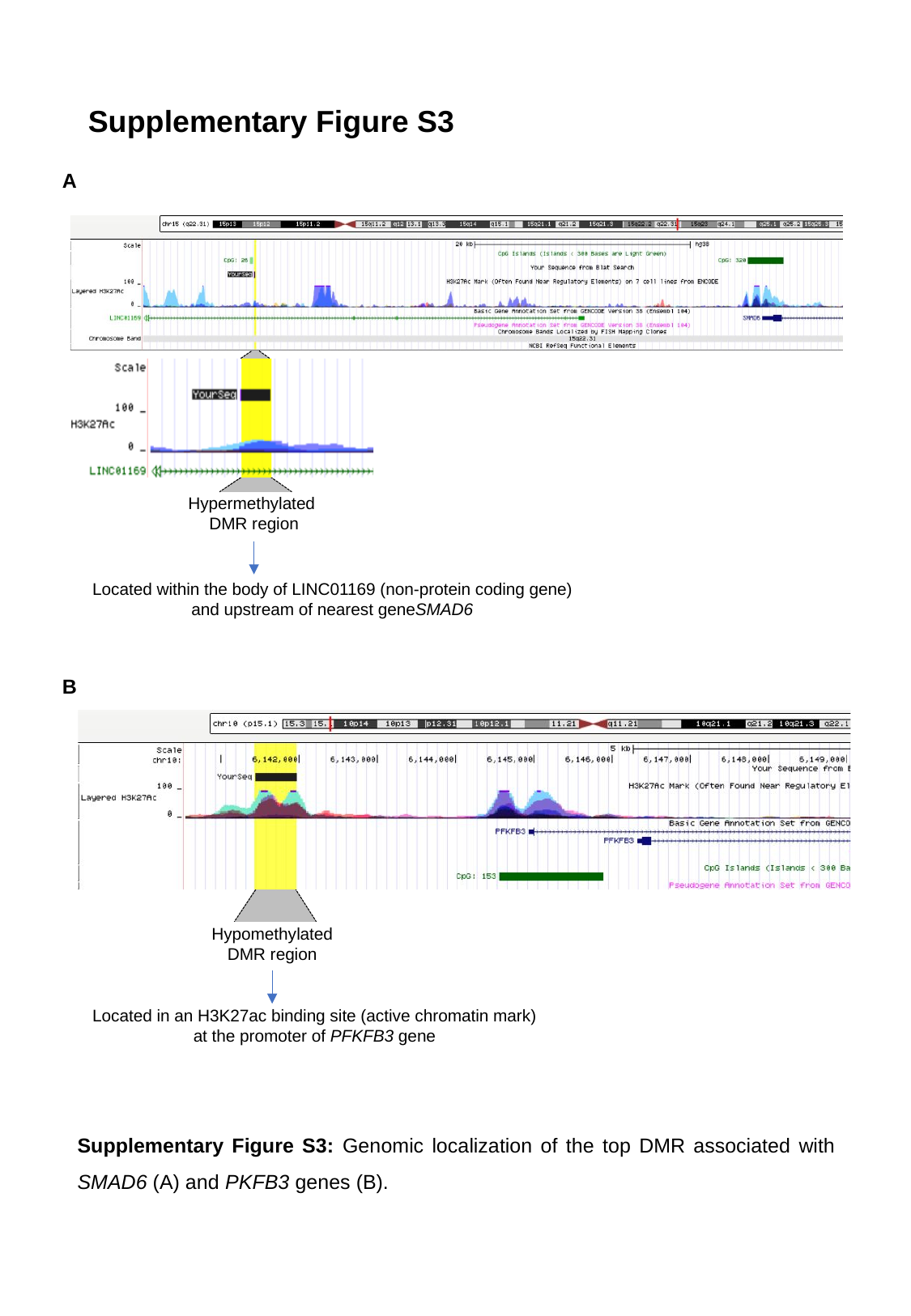

Supplementary Figure S3
A
Hypermethylated DMR region
Located within the body of LINC01169 (non-protein coding gene) and upstream of nearest geneSMAD6
B
Hypomethylated DMR region
Located in an H3K27ac binding site (active chromatin mark) at the promoter of PFKFB3 gene
Supplementary Figure S3: Genomic localization of the top DMR associated with SMAD6 (A) and PKFB3 genes (B).
